# Exploring the mechanistic role of alloying elements in copper-based electrocatalysts for the reduction of carbon dioxide to methane

**DOI:** 10.3389/fchem.2023.1235552

**Published:** 2023-08-07

**Authors:** Mingzhong Hao, Baorong Duan, Guorui Leng, Junjie Liu, Song Li, Shanshan Wang, Jiale Qu

**Affiliations:** ^1^ School of Rehabilitation Medicine, Binzhou Medical University, Yantai, China; ^2^ Research Center for Leather and Protein of College of Chemistry and Chemical Engineering, Yantai University, Yantai, China; ^3^ Department of Physics, Binzhou Medical College, Yantai, China; ^4^ School of Pharmacy (School of Enology), Binzhou Medical College, Yantai, China

**Keywords:** first-principles calculations, electrochemical CO_2_ reduction reaction, alloying effect, In–Cu alloy catalyst, overpotential

## Abstract

The promise of electrochemically reducing excess anthropogenic carbon dioxide into useful chemicals and fuels has gained significant interest. Recently, indium–copper (In–Cu) alloys have been recognized as prospective catalysts for the carbon dioxide reduction reaction (CO_2_RR), although they chiefly yield carbon monoxide. Generating further reduced C_1_ species such as methane remains elusive due to a limited understanding of how In–Cu alloying impacts electrocatalysis. In this work, we investigated the effect of alloying In with Cu for CO_2_RR to form methane through first-principles simulations. Compared with pure copper, In–Cu alloys suppress the hydrogen evolution reaction while demonstrating superior initial CO_2_RR selectivity. Among the alloys studied, In_7_Cu_10_ exhibited the most promising catalytic potential, with a limiting potential of −0.54 V versus the reversible hydrogen electrode. Analyses of adsorbed geometries and electronic structures suggest that this decreased overpotential arises primarily from electronic perturbations around copper and indium ions and carbon–oxygen bond stability. This study outlines a rational strategy to modulate metal alloy compositions and design synergistic CO_2_RR catalysts possessing appreciable activity and selectivity.

## 1 Introduction

Electrochemical reduction of CO_2_ into fuels is a promising measure to mitigate global warming and address the energy crisis (James et al., 2006; Schwartz, 2008; Handoko et al., 2018a; Bushuyev et al., 2018; De Luna et al., 2019; BhosaleRashid, 2022; Kang et al., 2023). One of the core technologies in this transformation process is the application of efficient electrocatalysts. However, existing catalysts are still unable to meet the requirements for commercial applications because of their high overpotentials and low partial current densities (Colin et al., 2012; Luo et al., 2022; Timoshenko et al., 2022). Hori et al. (1989) performed pioneering studies on the carbon dioxide reduction reaction (CO_2_RR), in which most of the transition metal catalysts are studied. In fact, only Cu metal is capable of producing large amounts of hydrocarbons from CO_2_, but it remains inefficient and requires a large overpotential (Calle-Vallejo and Koper, 2013; Ledezma-Yanez et al., 2016; Garza et al., 2018). Inevitably, the hydrogen evolution reaction (HER) competes with the CO_2_RR, resulting in reduced reaction efficiency and selectivity. In particular, the HER occurs at a limiting potential of approximately −0.5 V (*versus* a reversible hydrogen electrode (RHE); all potentials in this paper are *versus* RHE unless otherwise specified), but CH_4_ only starts to form at potentials of approximately −0.8 V (Hori et al., 1989; Hori, 2008). In fact, a potential of −1.0 V is required for a decent current density (to CH_4_) of 2 mA cm^-2^. Therefore, the design of highly active catalysts remains a major challenge in this field. As the process involves complex multi-electron and proton coupling steps, tuning the catalyst selectivity is equally, if not more, challenging.

Studies have proposed that alloying Cu with other metals, such as Au, Pd, and Sn, is an effective approach to enhancing CH_4_ selectivity at a lower overpotential (Hansen et al., 2016; Lee et al., 2018; Tran and Ulissi, 2018; Xie et al., 2018). Peterson and Nørskov (2012) predicted theoretically that *CO (* represents a surface adsorption species) cannot be stably adsorbed on a single metal surface and that a second metal with stronger oxygen affinity is needed to enable adsorption of both *CO and *CHO at suitable potentials. To this end, alloy catalysts are believed to produce CH_4_ at high selectivity by changing the binding strength of the key intermediates, such as *COOH, *OCHO, *CO, *CHO, and *COH (Nie et al., 2016; Nie et al., 2018). For example, Rodriguez et al*.* showed that Au@Cu nanoparticles with well-defined surface structures can selectively reduce CO_2_ to CH_4_, but the H_2_ formation increases with the number of Cu metal layers (Monzo et al., 2015). Gong *et al.* studied Pd–Cu alloy catalysts with different structures and suggested that Cu_3_Pd can decrease the onset potential for CH_4_ by 200 mV and show a seven-fold increase in CH_4_ current density at −1.2 V (Zhu et al., 2018). Recently, Cui et al*.* found electron donation from Sn to Cu in CuSn_3_ alloy catalysts, resulting in Sn^δ+^ oxidation states consistent with theory, as well as significantly improved selectivity of 95% at −0.5 V (Zheng et al., 2019).

Indium, as an *sp*-block metal, exhibits completely different catalytic properties compared to Cu metal. The CO_2_RR on In metal mainly produces large amounts of HCOOH and CO, in addition to showing strong inhibition to the HER (Bitar et al., 2016; Luo et al., 2019). However, the formation of HCOOH requires a potential of −1.8 V to achieve the highest current efficiency (Hori et al., 1989). These studies indicate that the bonding strengths of In and Cu metals to intermediate species such as *COOH and *OCHO are different. Takanabe *et al.* synthesized Cu–In alloy surfaces for electrochemical reduction of CO_2_ from mixed metal oxides of CuInO_2_ as the starting material. The sample was found to remarkably improve the selectivity of the CO_2_ reduction to form CO and formic acid, with a total FE of 94% for CO_2_ conversion (Jedidi et al., 2015). Giovanni et al. found that the potential range of the electrochemical reduction of CO_2_ at Cu–In alloy electrocatalysts of some compositions is −0.8 to −1.1 V vs. RHE (Hoffman et al., 2017). Andreas et al*.* studied that with a thin layer of metallic In deposited on the surface of the Cu nanowires, the catalyst exhibits a CO Faradaic efficiency of ∼93% at −0.7 to −0.9 V vs. RHE (Luo et al., 2018). It can be seen that In–Cu alloys have also been explored for CO_2_RR, and they are mostly found to produce CO as the predominant reduction product, and the overpotential range is high (Jedidi et al., 2015; Hoffman et al., 2017; Luo et al., 2018; Barasa et al., 2019). The formation of further reduced C_1_ products, such as CH_4_, remains elusive. This is because the alloying effect of In and Cu metals is not well understood, leading to difficulties in controlling the In–Cu alloy composition to fine-tune the catalyst activity and selectivity. Overall, theoretical studies of the synergistic alloying effect in In–Cu catalysts for the CO_2_RR are still lacking.

In the present work, the alloying effect of In with Cu metal is systematically investigated through first-principles calculations. We explore CO_2_RR properties over Cu and In metals and four different In–Cu alloys from the perspective of thermodynamics and kinetics. It is found that the introduction of In can improve the selectivity of the catalyst and reduce the overpotential for CO_2_RR. The alloying of In and Cu metals provides Cu-based catalysts superior selectivity in the initial stage of the CO_2_RR. For subsequent hydrogenation of *CO, the In–Cu alloys also exhibit a lower overpotential than Cu metal. Among these four different alloys, In_7_Cu_10_ possesses the least negative CO_2_RR limiting potential of −0.54 V. At the same time, the competing HER is significantly suppressed due to weak binding with H atoms. In order to clarify such a catalytic effect, the atomic adsorption configuration, the bonding characteristics, and the electron transfer of intermediate species were systematically analyzed by Bader charge, partial density-of-state (PDOS), and charge density analysis. The results indicate that the introduction of In metal changes the stability of intermediates and the electronic structure around Cu metal, thereby improving the catalytic activity significantly.

## 2 Methods and modeling

In this work, the first-principles calculations are performed based on density functional theory (DFT) as implemented in the Vienna *Ab initio* Simulation Package (VASP) code (Kresse and Furthmüller, 1996; Hafner and Kresse, 1997; Kresse and Joubert, 1999). The projector augmented wave (PAW) pseudopotential was adopted, and the generalized gradient approximation (GGA), as parametrized by Perdew–Burke–Ernzerhof (PBE) (Perdew et al., 1993; Perdew et al., 1996; Perdew et al., 1998), was used to treat the exchange-correlation effects of electrons. All simulations use a cutoff energy of 500 eV. During structure relaxation, the maximum force convergence accuracy acting on each atom is 0.01 eV/Å, and the total energy error is not more than 1.0 × 10^−4^ eV. To eliminate the interaction of the atoms between the slabs and avoid the interference of periodic alignment, the vacuum is set as large as 15 Å. The Brillouin zone was sampled using a Monkhorst–Pack k-point mesh of 5 × 5 × 1 for the 3 × 3 × 1 supercell. We also included the van der Waals (vdW) corrections by applying the vdW-DF2 method to all the systems. In this work, the climbing image-nudged elastic band (CI-NEB) method (Henkelman et al., 2000; Henkelman and Jónsson, 2000) is applied to search for the transition state and calculate the activation barrier of the reaction.

The CO_2_RR involves a multi-proton transfer step that produces many intermediate species (Handoko et al., 2018b; Handoko et al., 2019). Here, we focused on the catalytic effect of Cu metal and four different In–Cu alloys from the phase diagram, namely, In_3_Cu_7_, In_4_Cu_9_, InCu_2_, and In_7_Cu_10_. We optimized the bulk structure of the four different alloys and then cut the different crystal facets according to different Miller indices. Each slab is a five-layer structure, with the bottom three layers fixed and the others fully relaxed. We selected the close-packed facet of each alloy with enough stability and more active sites. The [111] facet of Cu metal, the [111] facet of In metal, the [2-32] facet of In_3_Cu_7_, the [01–1] facet of In_4_Cu_9_, the [100] facet of InCu_2_, and the [0-10] facet of In_7_Cu_10_ were adopted for the modeling simulation. In order to search for the most stable adsorption configuration of intermediate species, the lowest binding energies of 16 different adsorbed species, *COOH, *OCHO, *CO, *CHO, *COH, *H, *CH, *CH_2_, *CH_3_, *C, *O, *OH, *CH_2_O, *OCH_3_, *CHOH, and *CH_2_OH, are calculated. Herein, the binding energies are calculated using the following equation (Chuan et al., 2014):

$$E_{bind,C_{x}H_{y}O_{z}}=E_{slab+C_{x}H_{y}O_{z}}-\left(E_{slab}+xE_{f,C}+yE_{f,H}+zE_{f,O}\right),$$

where $E_{slab+C_{x}H_{y}O_{z}}$ represents the electronic energy of the slab–adsorbate complex minus the electronic energy of the clean slab and the formation energies of C, H, and O. The formation energy can be obtained by linearly combining the gas-phase electron energies of CO, H_2_O, and H_2_.

In this work, we adopted the computational hydrogen electrode (CHE) model to simulate the hydrogenation process of electrochemical reduction of CO_2_. In the CHE model, the free energy of each adsorbate along a reaction pathway that involves coupled proton–electron transfers is calculated using the following equation (Nørskov et al., 2004):

$$∆G_{n}\left(U\right)=∆G_{n}\left(U=0\right)+neU,$$

where n is the number of protons and electrons transferred and U is the potential *versus* the RHE. $∆G_{n}\left(U=0\right)$ is the free energy at 0 V *versus* the RHE. It can be calculated as the free energy of an equivalent hydrogenation step, where the chemical potential of each proton–electron pair can be replaced by the chemical potential of H_2_ at 1 bar because protons and electrons are in equilibrium with H_2_ at 1 bar at 0 V. To calculate this, zero-point energy and entropic corrections through vibrational frequencies are also simulated, which can be achieved by the harmonic approximation.

## 3 Results and discussion

In order to clarify the effect of In–Cu alloy structure on CH_4_ synthesis, we investigated the inhibition of the competitive HER, adsorption configuration, and electronic structure of key intermediates, as well as the energy profiles associated with CO_2_ hydrogenation on Cu metal, In metal, and four different In–Cu alloys. Since the initial hydrogenation of CO_2_ determines whether it can be reduced to hydrocarbons, we study this stage from the perspective of thermodynamics and kinetics. Finally, the effect of alloying on the limiting potential for hydrogenation of CO_2_ to CH_4_ is analyzed.

### 3.1 Effect of In–Cu alloy structure on the initial hydrogenation of CO_2_

Before investigating the CO_2_RR catalytic activity, the adsorption capacities for H atoms on these six different catalysts are considered in order to investigate the HER activity. The free energy for the HER was also calculated and is plotted in Supplementary Figure S9. The limiting potentials for HER on In–Cu alloys range from −0.71 V to −0.63 V, much more negative than that on Cu metal (−0.38 V). In metal shows the strongest inhibition effect on HER, on which the *H adsorption needs to overcome an energy of 0.81 eV. Thus, the In–Cu alloys are predicted to be less active than Cu metal for the HER, clearly indicating that the competitive HER can be efficiently suppressed on alloy surfaces.

**FIGURE 1:**
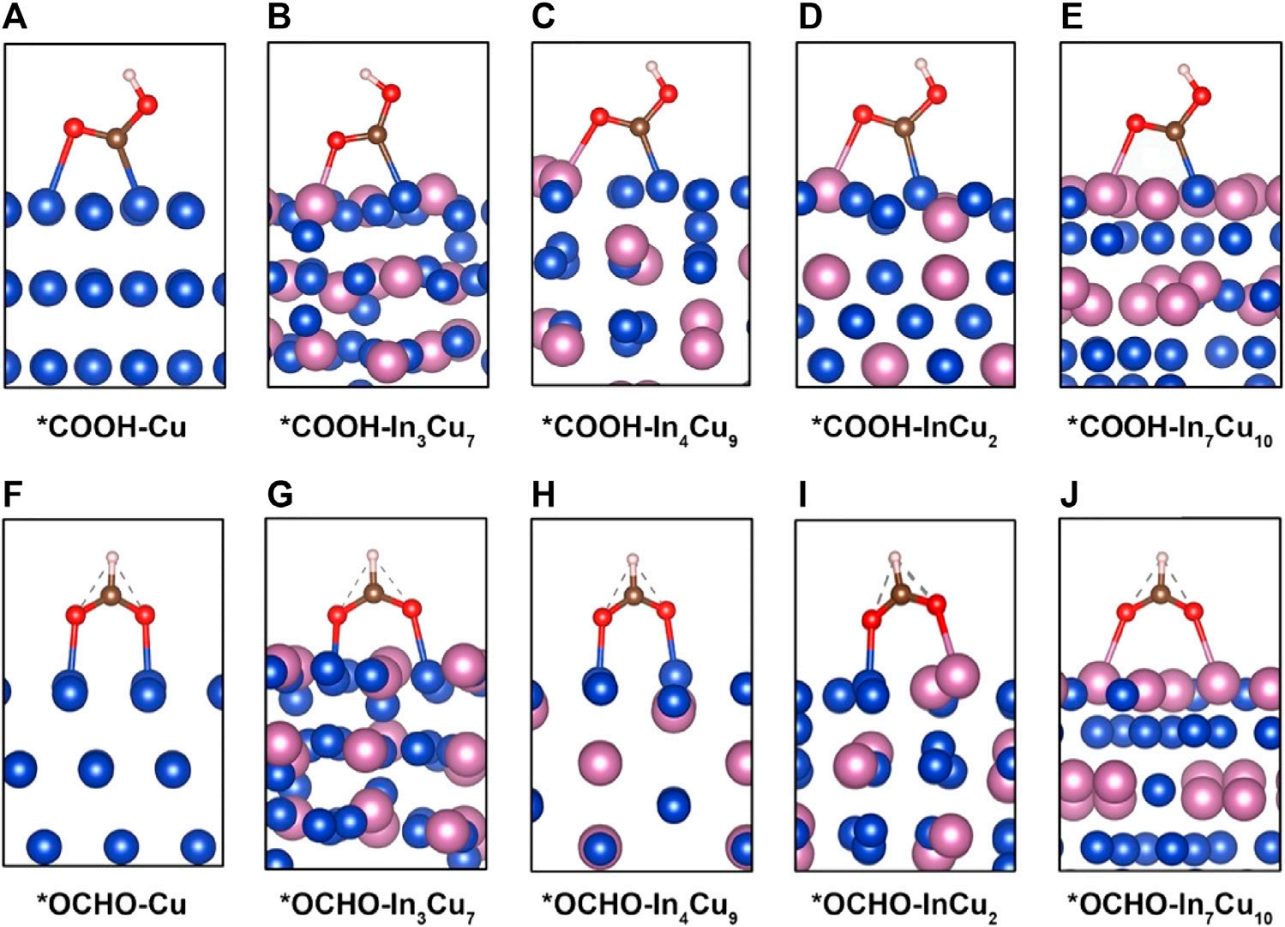
Adsorption configuration of intermediate *COOH on **(A)** Cu metal, **(B)** In_3_Cu_7_, **(C)** In_4_Cu_9_, **(D)** InCu_2_, and **(E)** In_7_Cu_10_. Adsorption configuration of intermediate *OCHO on **(F)** Cu metal, **(G)** In_3_Cu_7_, **(H)** In_4_Cu_9_, **(I)** InCu_2_, and **(J)** In_7_Cu_10_.

Continuously, we investigated the effect of In–Cu alloy structure on the initial hydrogenation of CO_2_ to *COOH and *OCHO intermediates. The energy profiles associated with these elementary steps are plotted in Figure 1. The Gibbs free energy diagrams for CO_2_-to-*CO and CO_2_-to-HCOOH can be calculated using the following reaction steps:

$${CO}_{2}+\left(H^{+}+e^{-}\right)\to *COOH$$

$$*COOH+\left(H^{+}+e^{-}\right)\to *CO+H_{2}O$$

$${CO}_{2}+\left(H^{+}+e^{-}\right)\to *OCHO$$

$$*OCHO+\left(H^{+}+e^{-}\right)\to HCOOH$$

**FIGURE 2:**
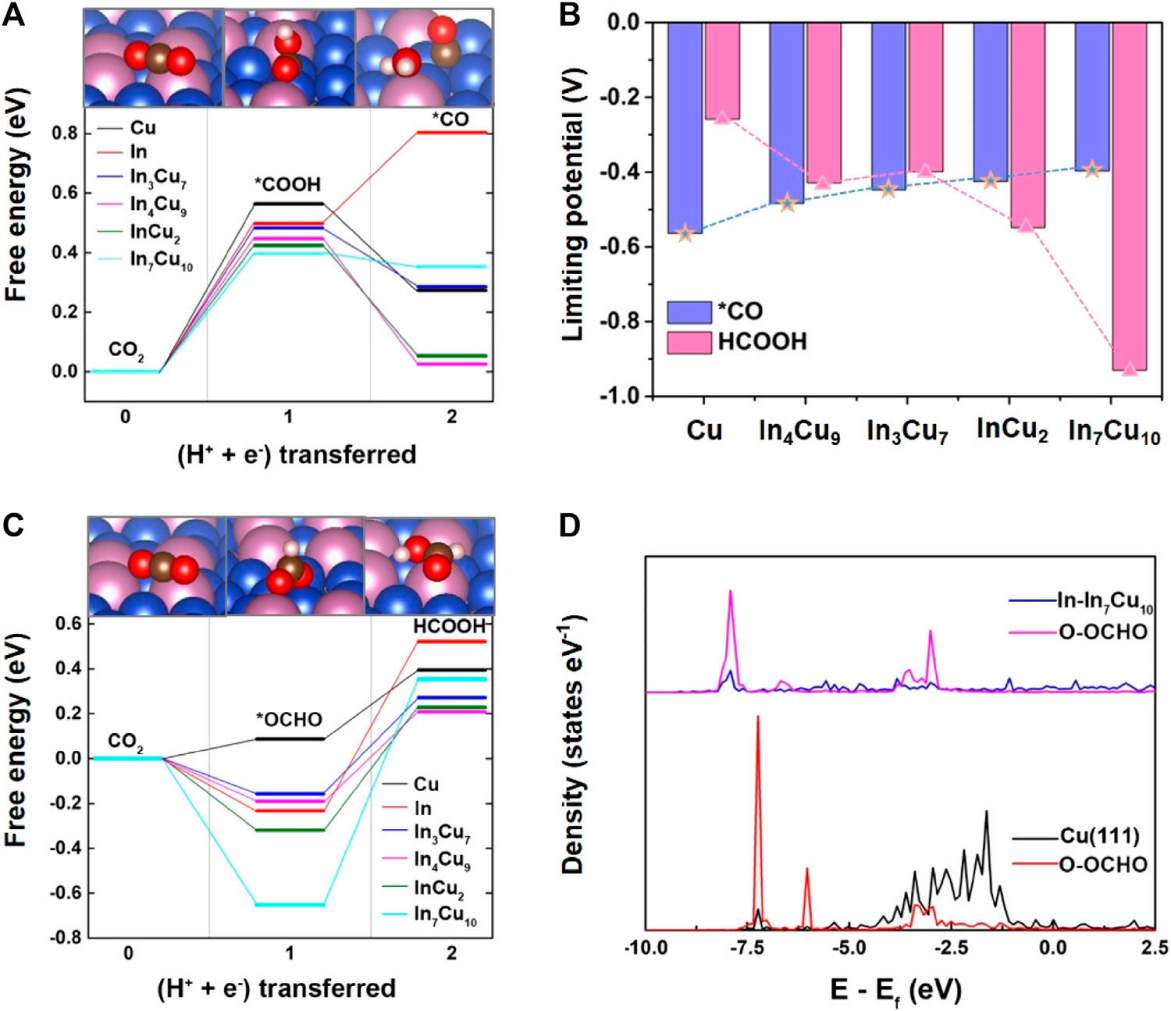
**(A)** Free energy diagrams for the lowest energy pathways to *CO for Cu metal, In metal, In_3_Cu_7_, In_4_Cu_9_, InCu_2_, and In_7_Cu_10_. **(B)** Limiting potential required for CO_2_-to-*CO and CO_2_-to-HCOOH on Cu metal, In_3_Cu_7_, In_4_Cu_9_, InCu_2_, and In_7_Cu_10_. **(C)** Free energy diagrams for the lowest energy pathways to HCOOH for Cu metal, In metal, In_3_Cu_7_, In_4_Cu_9_, InCu_2_, and In_7_Cu_10_. **(D)** Density of states of the intermediate *OCHO adsorbed on the Cu and In_7_Cu_10_ slabs.

Here, the asterisk (*) represents a surface adsorption species or an empty adsorption site. As shown in Figure 2A, *COOH is found to be the key reaction intermediate for CO_2_-to-*CO. Moreover, the required reaction energy for this step on Cu metal (0.56 eV) is larger than that on In–Cu alloys. Particularly, In_7_Cu_10_ requires the lowest reaction energy of only 0.39 eV. For In metal, although the reaction energy for CO_2_-to-*COOH is smaller than that for Cu metal (0.49 eV), the further hydrogenation of *CO to *CHO or *COH becomes difficult to occur because of the much higher reaction energy of 1.6 eV. Therefore, catalyzing CO_2_ to hydrocarbons using In metal is rather difficult (Jitaru et al., 1997).

As a competitive reaction, the reaction of CO_2_-to-*OCHO is also studied. In the pursuit of unraveling the intricate behavior of catalytic systems, a noteworthy discovery has been made with regard to the stable bidentate *OCHO configuration. This configuration arises from a fascinating interplay between the C atom and the H atom, resulting in the bonding of two O atoms to the surface of the catalyst (as illustrated in Figure 1). To further probe the energetic landscape of this catalytic phenomenon, the Gibbs free energy associated with the conversion of CO_2_ to HCOOH has been meticulously calculated (as demonstrated in Figure 2C). A compelling observation emerges, revealing that the reaction energy for CO_2_-to-*OCHO on In metal and its distinct amalgamations with Cu is negative. Intriguingly, on the sole Cu metal substrate, this energy is measured to be a modest 0.1 eV. Such discerning disparities in energy profiles underscore the thermodynamic favorability of *OCHO formation on the aforementioned alloys. Drawing parallels to the adsorption of *COOH, it becomes apparent that the stability of the *OCHO adsorption configuration escalates in tandem with the proportion of In atom content within the alloy. This observation highlights the synergistic effects between In and Cu atoms in promoting enhanced stability within the catalytic system. Figure 2B shows the different trends of limiting potential for *CO and HCOOH on these five different catalysts, indicating that the introduction of In metal can result in stronger selectivity at the initial hydrogenation stage of CO_2_.

It can be rationally predicted that the distinct CO_2_RR activity on In–Cu alloys can be attributed to the introduction of In metal. In order to explicate this phenomenon, the adsorption configurations of *COOH and *OCHO species on the surfaces of five distinct catalysts (Cu, In_3_Cu_7_, In_4_Cu_9_, InCu_2_, and In_7_Cu_10_) are visually depicted in Figure 1. Within the context of Cu metal, both the C atom and the O atom in the *COOH moiety exhibit an affinity toward bonding with the Cu atom. Conversely, upon considering the In–Cu alloy catalysts, the O atom evinces a proclivity to establish a bond with the In atom, while the C atom persists in exhibiting an inclination to bond with the Cu atom. This amalgamation of bonding modalities engenders a hybridized bonding mode, thereby conferring enhanced stability upon the adsorption of the *COOH species. This increased stability can be further explicated and corroborated through the employment of partial density-of-state (PDOS) analysis, as denoted in Supplementary Figure S1. On Cu metal, the energy level of bonding between the O atoms in the *COOH and the Cu atoms on the surface is −7.5 eV, while the bonding between the O and In atoms on all of the In–Cu alloys shifts to a lower energy level. For In_3_Cu_7_, In_4_Cu_9_, InCu_2_, and In_7_Cu_10_, the typical bonding peaks correspond to the energies of −7.7 eV, −8.4 eV, −8.0 eV, and −8.4 eV, respectively. It is worth noting that the introduction of In metal cannot destroy the bonding between C and Cu atoms. Thus, this mixed bonding mode induced by In–Cu alloys is beneficial to the stability of *COOH. A similar effect also exists on the surface adsorption of the *OCHO intermediate. As shown in Supplementary Figure S1, both O atoms in *OCHO forms a bond with Cu atoms on the surfaces of Cu, In_3_Cu_7_, and In_4_Cu_9_ catalysts but forms a bond with In atoms on the In_7_Cu_10_ catalyst. It can also be seen from the PDOS profiles (Figure 2D) that the energy level of higher hybridization between the O atom and Cu atom is −7.2 eV, while the energy level of hybridization between the O atom and In atom on the In_7_Cu_10_ surface shifts to −8 eV. The lower level indicates that the formation of the O–In bond is more favorable for *OCHO on the In–Cu alloy catalysts.

**FIGURE 3:**
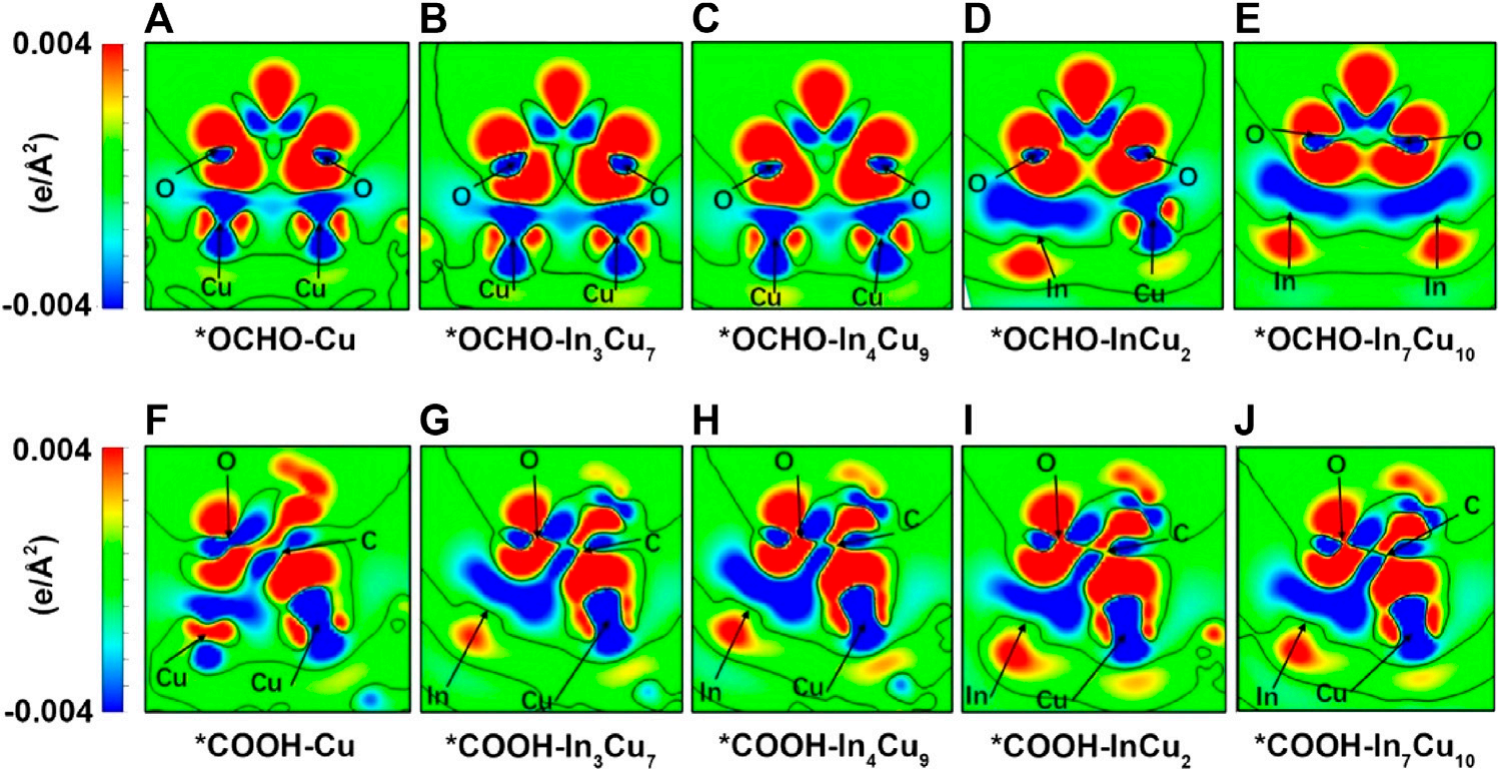
Charge density difference (the difference between the charge spatial distribution before and after species adsorption. The isosurface level is set to 1.49911 e∙nm^-3^) for *OCHO on **(A)** Cu metal, **(B)** In_3_Cu_7_, **(C)** In_4_Cu_9_, **(D)** InCu_2_, and **(E)** In_7_Cu_10_. Charge density difference for *COOH on **(F)** Cu metal, **(G)** In_3_Cu_7_, **(H)** In_4_Cu_9_, **(I)** InCu_2_, **(J)** In_7_Cu_10_.

The Gibbs free energy changes (ΔG) are closely related to the charge transfer of key ions. In order to prove it, we calculated the charge density difference of the *COOH and *OCHO atomic layers for Cu metal and four different In–Cu alloy catalysts (Figure 3). Meanwhile, the Bader charges on the key O and C ions in various intermediates have also been quantitatively calculated. We calculated the Bader charges in the initial state *CO_2_ (*e*_ini_) and final state *COOH/*OCHO (*e*_fin_) and focused on the difference between them (Δ*e* = *e*_fin_ − *e*_ini_). The Δ*e* values for both CO_2_-to-*COOH and CO_2_-to-*OCHO reaction steps are summarized in Table 1. For *OCHO, the charge change in the O ions is considered to be closely related to ΔG. As shown in Table 1, the O ion on Cu metal can only capture approximately 0.15 e from the Cu ion. Based on the results of the PDOS analysis of bonded Cu atoms at the surface and bulk Cu atoms, we found that their electronic structures show different distributions. The peaks of the *p*_*x*_ and *p*_*y*_ orbitals of Cu metal are significantly reduced around the Fermi level after CO_2_ adsorption, while the peaks of the *p*_*z*_ orbital shift to a high energy level (Supplementary Figure S3). At the same time, it can also be clearly observed from Figure 3A that a large amount of charge is transferred from the *p*_*z*_ to *p*_*x*_ orbit inside the Cu ion, which clearly indicates that the on-site orbit transfer dominates when the intermediate interacts with Cu metal. For In_3_Cu_7_ and In_4_Cu_9_, the electrons around Cu ions are still undergoing an orbital transfer (Figure 3B; c), but the Bader charge indicates that more electrons transfer to the O ion from the Cu ion. This proves that the introduction of In ions can inhibit the on-site electron orbital transfer on the Cu ion and promote electron migration to the O ion. For both InCu_2_ and In_7_Cu_10_ catalysts, the In ion gradually replaced the Cu ion to bond with *OCHO, inducing an increasing amount of charge around O ions (average 0.18 e). For the *COOH configuration, charge transfer follows a similar trend to *OCHO, and the introduction of In ions can also make the Cu ion lose more electrons. As the content of the In ion increases, the C and O ions in *COOH receive more charge, which is consistent with the trend of the ΔG for CO_2_-to-*OCHO (more and more negative). Therefore, it can be clearly seen that the In ion can not only enhance the binding strength of the CO_2_RR intermediate but also regulate the charge distribution around the Cu ions.

**TABLE 1:** Bader charges of C/O ion and Cu/In ion in different slabs. Δ*e* = *e*_ini_ − *e*_fin_ (where *e*_ini_ represents the charge of initial configuration and *e*_fin_ represents the final configuration).

System	ΔG		Δ*e*		Δ*e*
Cu-OCHO	0.08	O	0.08	Cu	−0.15
		O	0.07	Cu	−0.14
In_3_Cu_7_-OCHO	−0.15	O	0.12	Cu	−0.18
		O	0.10	Cu	−0.16
In_4_Cu_9_-OCHO	−0.19	O	0.13	Cu	−0.17
		O	0.12	Cu	−0.17
InCu_2_-OCHO	−0.31	O	0.12	Cu	−0.26
		O	0.19	In	−0.28
In_7_Cu_10_-OCHO	−0.65	O	0.17	In	−0.27
		O	0.16	In	−0.26
Cu-COOH	0.56	C	0.77	Cu	−0.14
		O	0.09	Cu	−0.11
In_3_Cu_7_-COOH	0.48	C	0.82	Cu	−0.14
		O	0.15	In	−0.25
In_4_Cu_9_-COOH	0.44	C	0.87	Cu	−0.16
		O	0.17	In	−0.25
InCu_2_-COOH	0.42	C	0.89	Cu	−0.16
		O	0.16	In	−0.26
In_7_Cu_10_-COOH	0.39	C	0.89	Cu	−0.21
		O	0.17	In	−0.26

**FIGURE 4:**
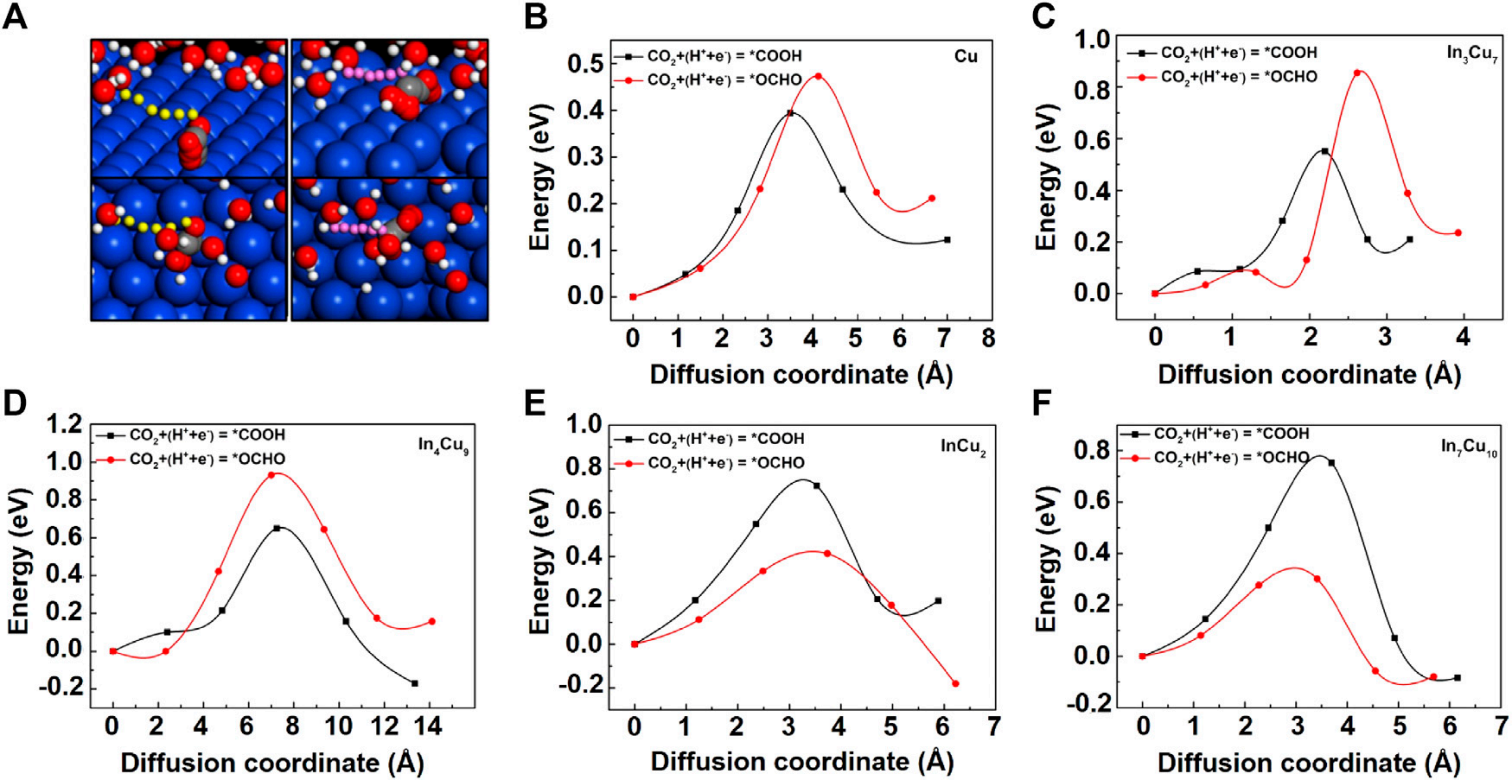
**(A)** The graph on the left shows CO_2_-to-*COOH on Cu metal, and the graph on the right shows CO_2_-to-*OCHO on Cu metal. The reaction pathways on the In–Cu alloys are the same as those on Cu metal. The six positions of the transferred proton (yellow on the left and pink on the right), the H atoms (white in H_2_O molecules), O atoms (red), and C atoms (gray), starting with the initial state and ending at the final state, are shown. The transition energy barrier for the CO_2_-to-*COOH and CO_2_-to-*OCHO reactions of **(B)** Cu metal, **(C)** In_3_Cu_7_, **(D)** In_4_Cu_9_, **(E)** InCu_2_, and **(F)** In_7_Cu_10_.

The kinetic properties of In–Cu alloys also play a decisive role in the efficiency of the CO_2_RR. Here, we adopt the CI-NEB scheme to compute the activation barrier of the initial key step: CO_2_-to-*COOH and CO_2_-to-*OCHO on Cu metal and four different In–Cu alloys, while the energy evolution profiles are shown in Figure 4. For Cu metal, the barriers for *COOH is 0.1 eV lower than that for *OCHO. Such a negligible advantage on the barrier cannot play a key role in the inhibition of HCOOH formation, which can be viewed as an important reason for its poor selectivity (Xie et al., 2016). This advantage has been expanded on In_3_Cu_7_ and In_4_Cu_9_. For In_3_Cu_7_, the barrier for *COOH is 0.3 eV lower than that for *OCHO, while for In_4_Cu_9_, the difference was also increased to 0.28 eV. However, such a reaction trend has been changed on InCu_2_ and In_7_Cu_10_. For InCu_2_, the barrier for *OCHO is 0.31 eV lower than that for *COOH. In addition, for In_7_Cu_10_, this difference is further expanded to 0.45 eV. Considering both aspects of thermodynamics and kinetics, we can clearly see that these four In–Cu alloys exhibit different selectivities for CO_2_-to-*CO and CO_2_-to-HCOOH. For In_3_Cu_7_ and In_4_Cu_9_, their thermodynamic-based pathway is consistent with the kinetic-based pathway, indicating that the alloying effect is more conducive to further hydrogenation of *CO. However, for InCu_2_ and In_7_Cu_10_, their kinetic-based pathways exhibit an opposite tendency to the thermodynamic-based pathways. According to the Erying equation $\left(k=\frac{k_{B}T}{h}K_{c}^{\neq },K_{c}^{\neq }=ALe^{-\frac{E_{0}}{RT}}\right)$ (Eyring, 1935), the hydrogenation reaction follows a thermodynamically favorable path (CO_2_-to-*CO) at a lower temperature. However, as the reaction temperature increases, the kinetically favorable path (CO_2_-to-HCOOH) takes a significant advantage. Despite this, the formation of HCOOH becomes still difficult on InCu_2_ and In_7_Cu_10_ due to the high overpotential requirements. Therefore, at mild reaction temperatures and lower negative reaction potentials, In–Cu alloys can exhibit better selectivity than Cu metal for CO_2_-to-*CO/CO_2_-to-HCOOH, which indicates that the In–Cu alloy is more promising to reduce CO_2_ to hydrocarbons.

### 3.2 Limiting potential for CO_2_ hydrogenation to CH_4_ over In–Cu alloys

**FIGURE 5:**
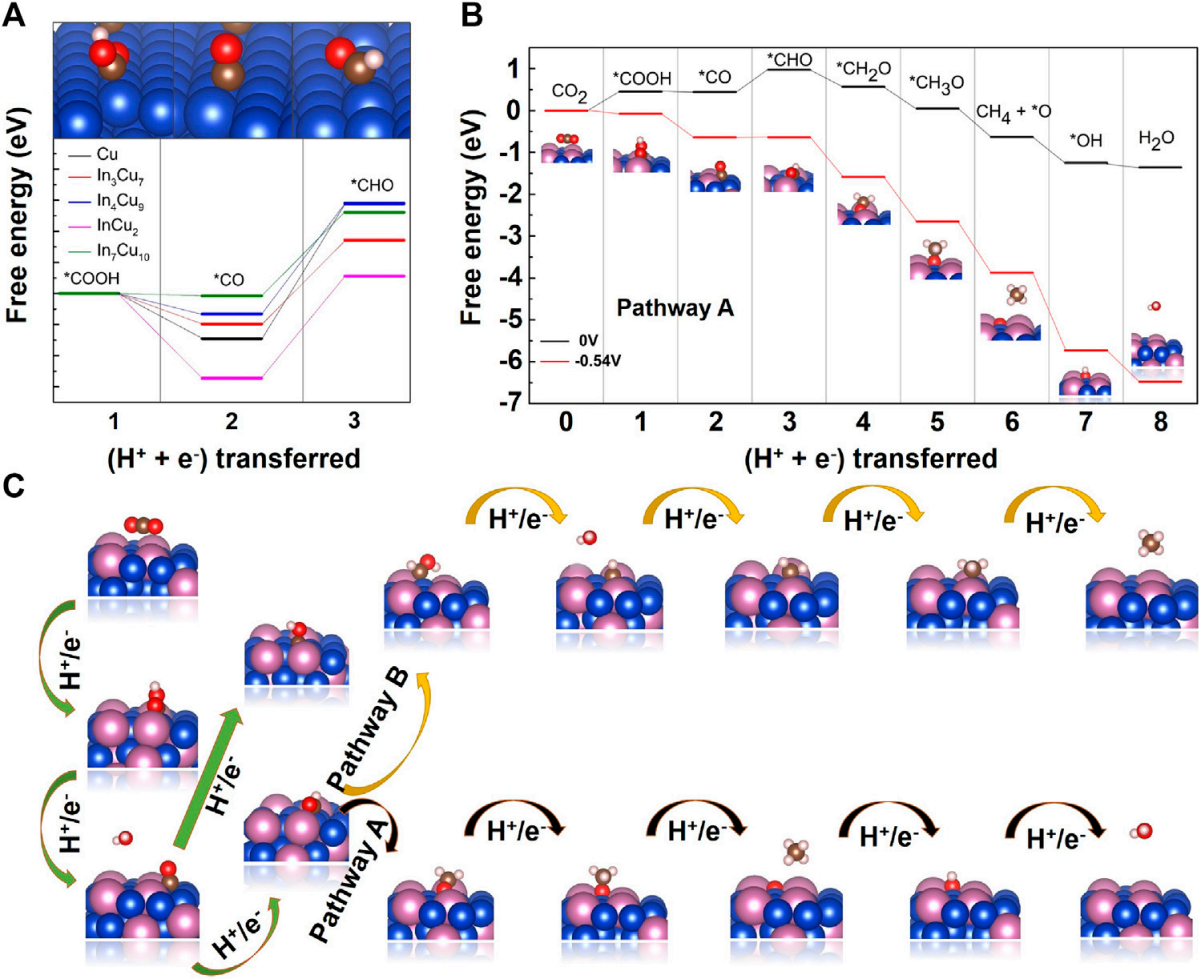
**(A)** Free energy diagrams for the lowest energy pathways to *CHO on Cu metal, In_3_Cu_7_, In_4_Cu_9_, InCu_2_, and In_7_Cu_10_. **(B)** Free energy diagrams for the lowest energy pathways to CH_4_ on In_7_Cu_10_. The pathway in black (higher) represents the free energy at 0 V vs. RHE, and the pathway in red (lower) represents the free energy at the indicated potential. **(C)**Two different pathways for hydrogenation of CO_2_ to CH_4_.

In order to investigate the limiting potential for CO_2_ hydrogenation to CH_4_ over In–Cu alloys, we calculated the complete Gibbs free energy phase diagram. Combining thermodynamics and kinetics, the optimal paths to CH_4_ on Cu metal and four different In–Cu alloys are determined (Figure 5, Supplementary Figures S4–S8). We define the pathways as pathway A: CO_2_ → *COOH → *CO → *CHO → *CH_2_O → *CH_3_O → CH_4_ + *O → *OH → H_2_O and pathway B: CO_2_ → *COOH → *CO → *CHO → *CHOH → *CH → *CH_2_ → *CH_3_ → CH_4_. Since *CH_2_O and *CH_3_O are not stable on the InCu_2_ catalyst, the optimal path for InCu_2_ is pathway A while that for the other three different In–Cu alloys is pathway B. We focus on the hydrogenation of *CO to *CHO, as it is a key step for further hydrogenation. As shown in Supplementary Figure S4, the reaction energy for this step on Cu metal is 0.87 eV, which means a relatively large limiting potential for the subsequent hydrogenation. The results (Supplementary Figures S5–S8) show that the reaction energies for In_3_Cu_7_, In_4_Cu_9_, InCu_2_, and In_7_Cu_10_ are 0.69 eV, 0.71 eV, 0.65 eV, and 0.54 eV, respectively. In particular, the limiting potential is smaller than 0.6 V on In_7_Cu_10_. The calculation results show that the In–Cu alloys have the potential to exhibit better catalytic performance for the CO_2_RR than Cu metal.

**FIGURE 6:**
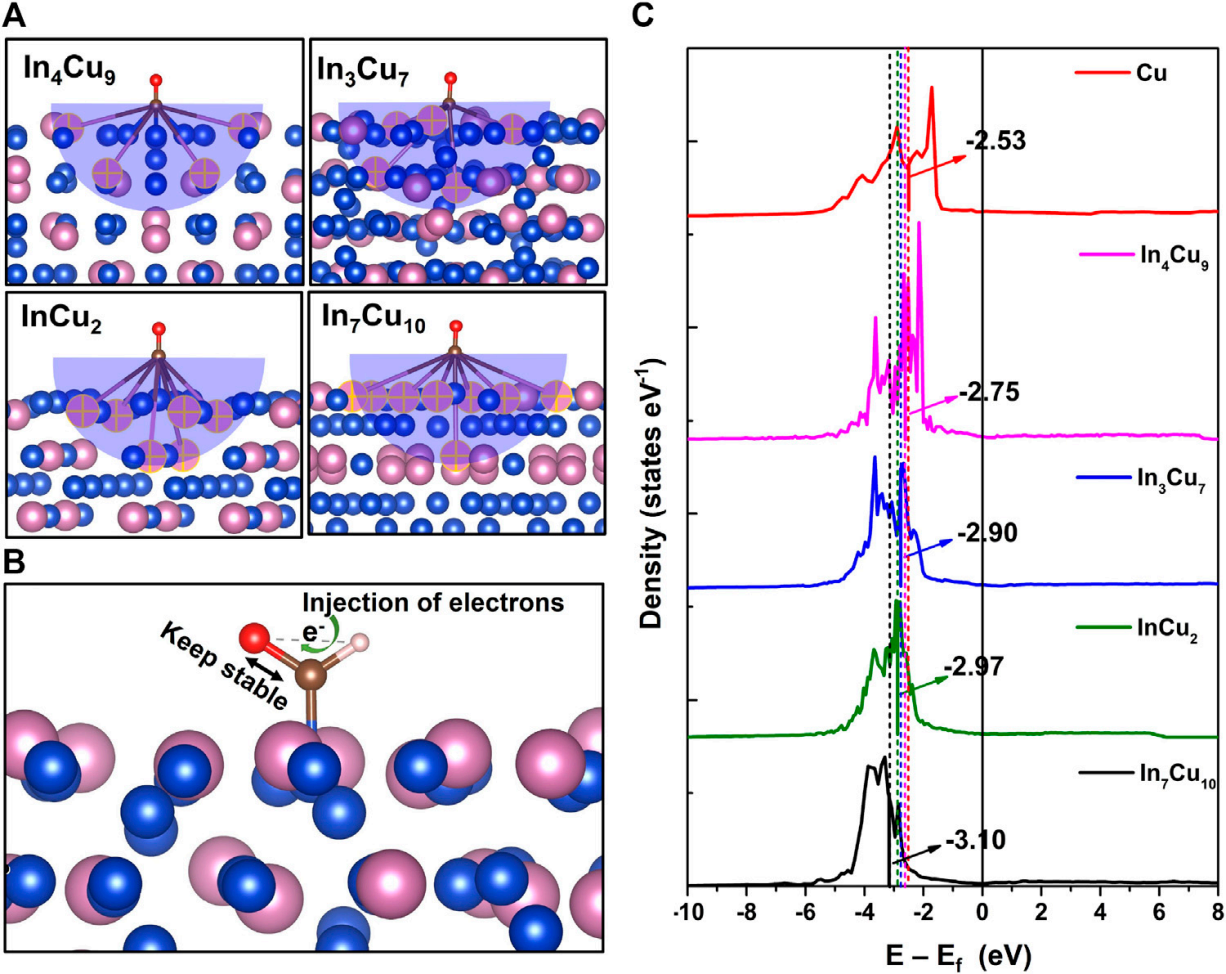
**(A)** Number of effective In atoms involved in the reaction determined by the Bader charge (marked by the yellow cross). **(B)** Schematic diagram of the H atom injecting electrons into C-O bonds to maintain C-O bond stability. **(C)** d-band center for the Cu atom in Cu metal, In_4_Cu_9_, In_3_Cu_7_, InCu_2_, and In_7_Cu_10_.

In order to analyze the mechanism of the reduced reaction energy for the key step, we performed Bader charge and bonding analysis on *CO and *CHO configurations. It can be reasonably predicted that only In atoms near the molecule can participate in the reaction of *CO to *CHO, so we first studied the effective interaction distance. We calculated the Bader charge of all ions in the slab before and after *CO adsorption on Cu metal, In_3_Cu_7_, In_4_Cu_9_, InCu_2_, and In_7_Cu_10_. Then, the effective radius of 5.5 Å can be determined around the adsorption site, where the charge change on In ions is greater than 0.1 e. The absolute value of the charge change is negligible out of this range. As shown in Figure 6A, the numbers of effective In ions in In_3_Cu_7_, In_4_Cu_9_, InCu_2_, and In_7_Cu_10_ are 5, 4, 6, and 8, respectively. At the same time, we counted the amount of charge on C-O pairs in *CO and *CHO and found that it remained almost the same during the process of *CO-to-*CHO. However, the Bader charge of H ions undergoes significant changes. For Cu metal, In_3_Cu_7_, In_4_Cu_9_, InCu_2_, and In_7_Cu_10_, the charge on H ions is 0.81 e, 0.90 e, 0.92 e, 0.95 e, and 0.97 e, respectively. Therefore, we predict that the C-O bond needs to be stable to ensure that it cannot be broken during further hydrogenation (*CO → *CHO → *CH_2_O or *CO → *CHO → *CHOH). The added H ions are responsible for injecting electrons into the C-O bond to maintain its stability, which is related to ΔG of the H adsorption process (Figure 6B). Furthermore, we extracted Cu atoms in the effective interaction range to calculate their d-band center, as shown in Figure 6C. It can be found that as the number of effective In atoms increases, the d-band center gradually shifts away from the Fermi energy level, indicating that the introduction of In metal not only regulates the charge transfer around Cu ions but also affects their d-band center. As a result, the dissociation effect from the *d* orbit electron to the C-O bond is weakened.

**FIGURE 7:**
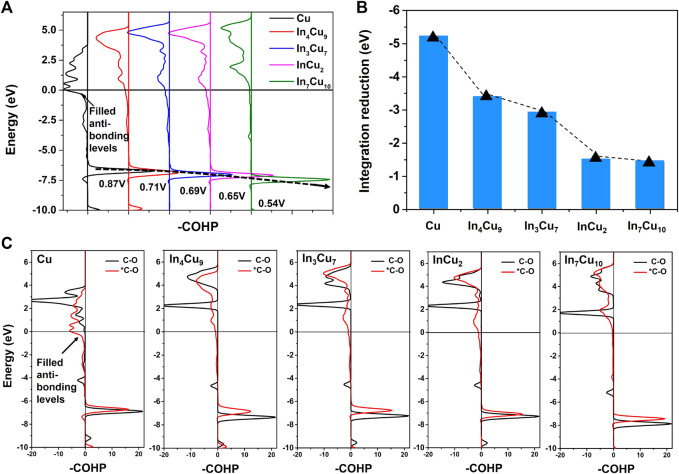
**(A)** -COHP for C-O bonds in *CO on Cu metal, In_4_Cu_9_, In_3_Cu_7_, InCu_2_, and In_7_Cu_10_. **(B)** Reduction of C-O bond integration after freestanding CO is adsorbed on five different catalyst surfaces. **(C)** -COHP for C-O bonds in CO and *CO on Cu metal, In_4_Cu_9_, In_3_Cu_7_, InCu_2_, and In_7_Cu_10_.

Based on the calculation results of the d-band center, we reasonably predict that the C-O bonds on all of the alloy catalysts are more stable than those on Cu metal. In order to confirm this, we further calculated the crystal orbital Hamilton population (COHP) (Deringer et al., 2011; Maintz et al., 2013; Maintz et al., 2016) for the C-O bond on Cu metal and four different In–Cu alloys, as shown in Figure 7A. When *CO is adsorbed on Cu metal, the anti-bonding level of the C-O bond will be partially filled, but on In–Cu alloys, such an anti-bond occupation effect becomes insignificant. It is worth noting that the bonding levels of the C-O bond gradually shift to lower energy regions in the order of Cu, In_4_Cu_9_, In_3_Cu_7_, InCu_2_, and In_7_Cu_10_, which is consistent with the trend of potential. Occupied anti-bond states and higher bonding states indicate that the C-O bond is more unstable on Cu metal than on In–Cu alloys. For comparison, we calculated the COHP for freestanding CO molecules in these five different catalysts, as shown in Figure 7C. The results confirm that the anti-bond state is occupied after the freestanding CO molecule is adsorbed on the catalyst surface. In order to characterize the destruction of the C-O bond by the catalyst surface, we calculated the bond state integration of the C-O bond before and after freestanding CO molecule adsorption and focused on the integration difference between them (Figure 7B). It can be seen that the destruction effect caused by the catalyst is also decreasing in the order of Cu, In_4_Cu_9_, In_3_Cu_7_, InCu_2_, and In_7_Cu_10_. Particularly for the In_7_Cu_10_ catalyst, the bonding state of C-O after CO molecule adsorption is almost the same as in the freestanding case. Therefore, we confirmed that the introduction of In metal will regulate the electronic structure around Cu metal, resulting in changeable stability of the surface intermediates and finally affecting the reaction energy of further hydrogenation.

## 4 Conclusion

In summary, we studied the catalytic properties of In–Cu alloys for the CO_2_RR and found that In–Cu alloys possess better selectivity for the initial stage of CO_2_RR and require less negative potential for CO_2_RR to form CH_4_. The In_7_Cu_10_ catalyst is found to be the most promising catalyst, with a limiting potential of −0.54 V. By deeply investigating the mechanism at the atomic level, it is found that In metal improves the selectivity by affecting the stability of the adsorption configuration. In addition, In metal can adjust the electron transfer between the intermediate species and Cu metal, thereby affecting the reaction energy. The introduction of In metal shifts the Cu d-band center to a more negative energy level, which can change the stability of *CO and facilitate further hydrogenation. As a result, compared with Cu metal, the In–Cu alloys can lead to completely different catalytic activity for CO_2_RR and effectively lower the overpotential required for CO_2_RR. Our study demonstrates the promise of In–Cu alloys in CO_2_RR and, at the same time, provides new insights into designing other synergistic metal alloy catalysts with high activity and selectivity.

## Data Availability

The original contributions presented in the study are included in the article/Supplementary Material; further inquiries can be directed to the corresponding authors.
