# Supplementary material for: Exploring the mechanistic role of alloying elements in copper-based electrocatalysts for the reduction of carbon dioxide to methane
Source: Front Chem. 2023 Aug 7;11:1235552. doi: 10.3389/fchem.2023.1235552 (PMC10440379; doi:10.3389/fchem.2023.1235552)
Supplement: Supplementary file 1 [file DataSheet1.docx]

# Supplementary Information

# Exploring the Mechanistic Role of Alloying Elements in Copper-Based Electrocatalysts for the Reduction of Carbon Dioxide to Methane

Mingzhong Hao^1^^,§^· Baorong Duan^2,§^ · Guorui Leng^3,§^ · Junjie Liu^4^ · Song Li^3^ · Shanshan Wang^5,^* · Jiale Qu^1,^*

*1. Institute of Rehabilitation Engineering, Binzhou Medical University, Yantai 264003, China*

*2. Research Center for Leather and Protein of College of Chemistry & Chemical Engineering, Yantai University, Yantai 264005, China*

*3. School of Rehabilitation Medicine, Binzhou Medical University, Yantai 264003, China*

*4. Department of Physics, Binzhou Medical Ccollege, Yantai 264003, China*

*5. School of pharmacy (School of Enology), Binzhou Medical Ccollege, Yantai 264003, China*

§Author contributions: These authors contributed equally to this work.

*corresponding author：qujiale@buaa.edu.cn; jwangshan@163.com


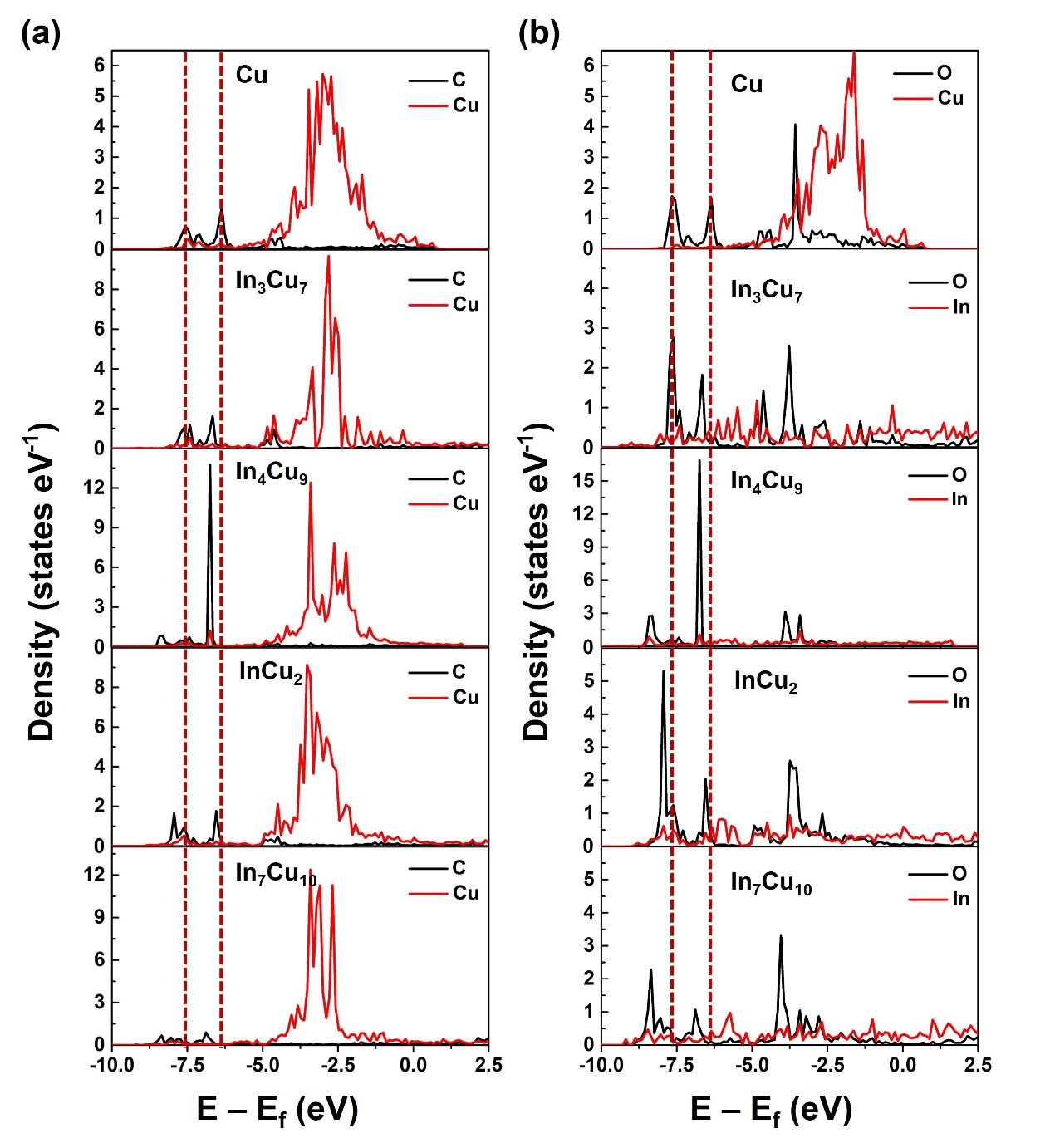


**Fig. S1.** Density of states of the intermediate *COOH adsorbed on Cu metal, In_3_Cu_7_, In_4_Cu_9_, InCu_2_ and In_7_Cu_10_. (a) Density of states of the C atom and Cu atom on the surface. (b) Density of states of the O atom and In atom on the surface (for Cu is density of states of the O atom and Cu atom).


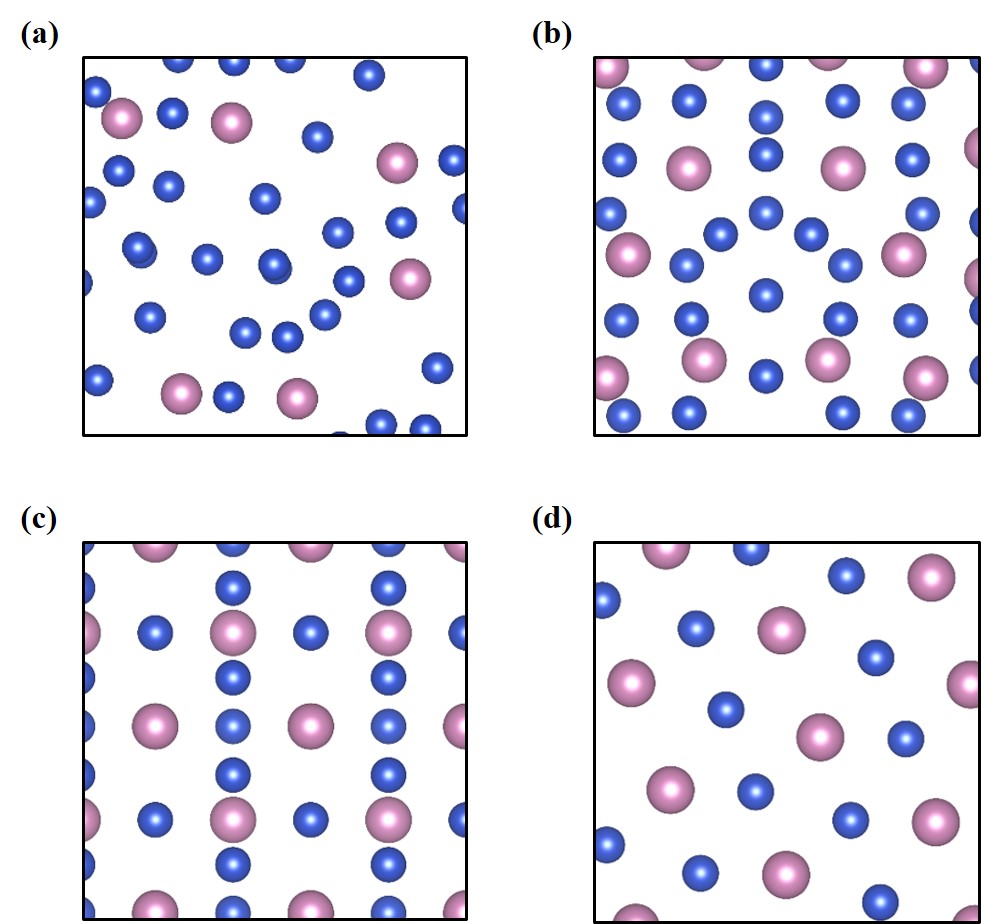


**Fig. S2.** The distribution of In atoms and Cu atoms on the surface of (a) In_3_Cu_7_, (b) In_4_Cu_9_, (c) InCu_2_ and (d) In_7_Cu_10_.


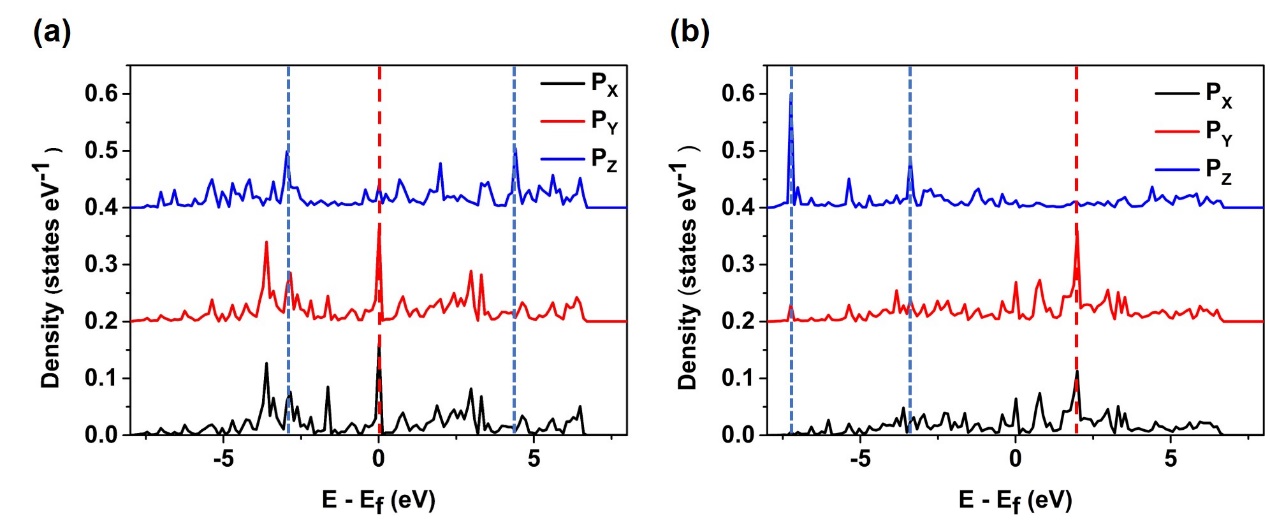


**Fig. S3.** The distribution of electron structure changed after Cu atoms bond with *OCHO. From PDOS, it can be seen that electrons transfer from p*_x_* and p*_y_* orbit to p*_z_* orbit.


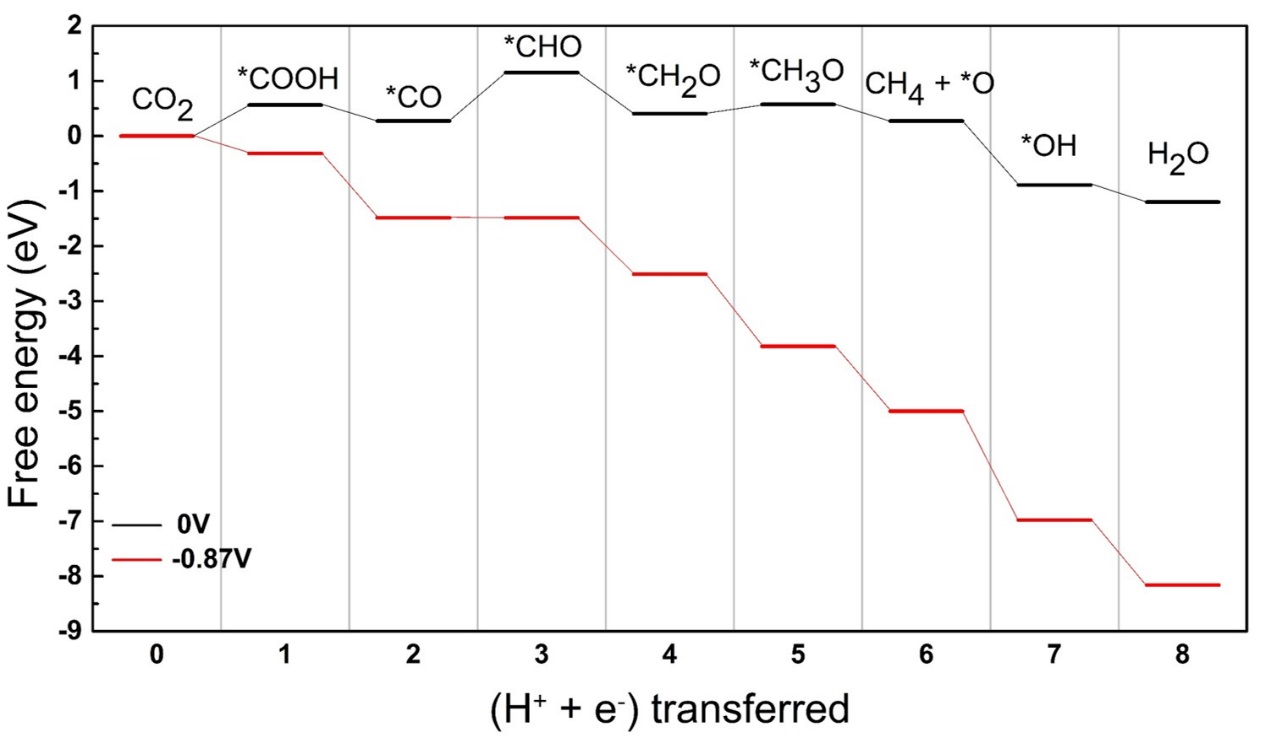


**Fig. S4.** Free energy diagrams for the lowest energy pathways to CH_4_ on Cu metal. The black (higher) pathway represents the free energy at 0 V *vs.* RHE and the red (lower) pathway the free energy at the indicated potential.


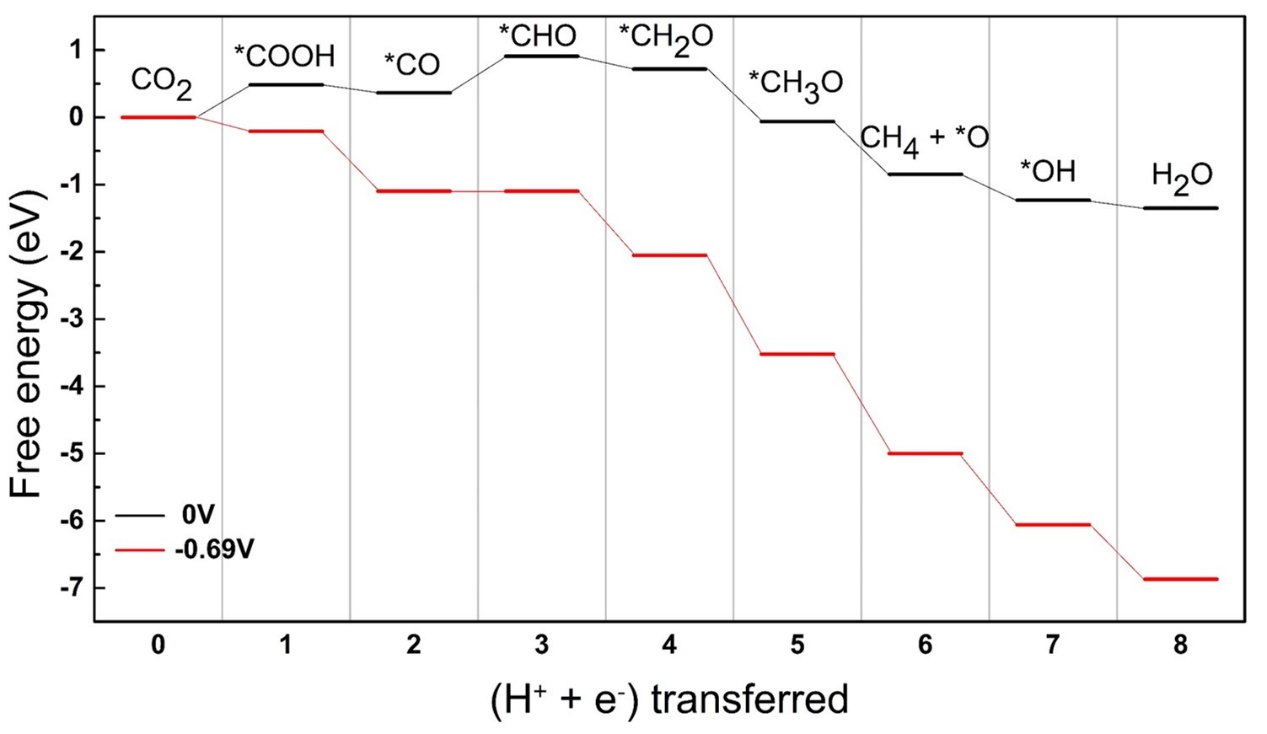


**Fig. S5.** Free energy diagrams for the lowest energy pathways to CH_4_ on In_3_Cu_7_. The black (higher) pathway represents the free energy at 0 V *vs.* RHE and the red (lower) pathway the free energy at the indicated potential.


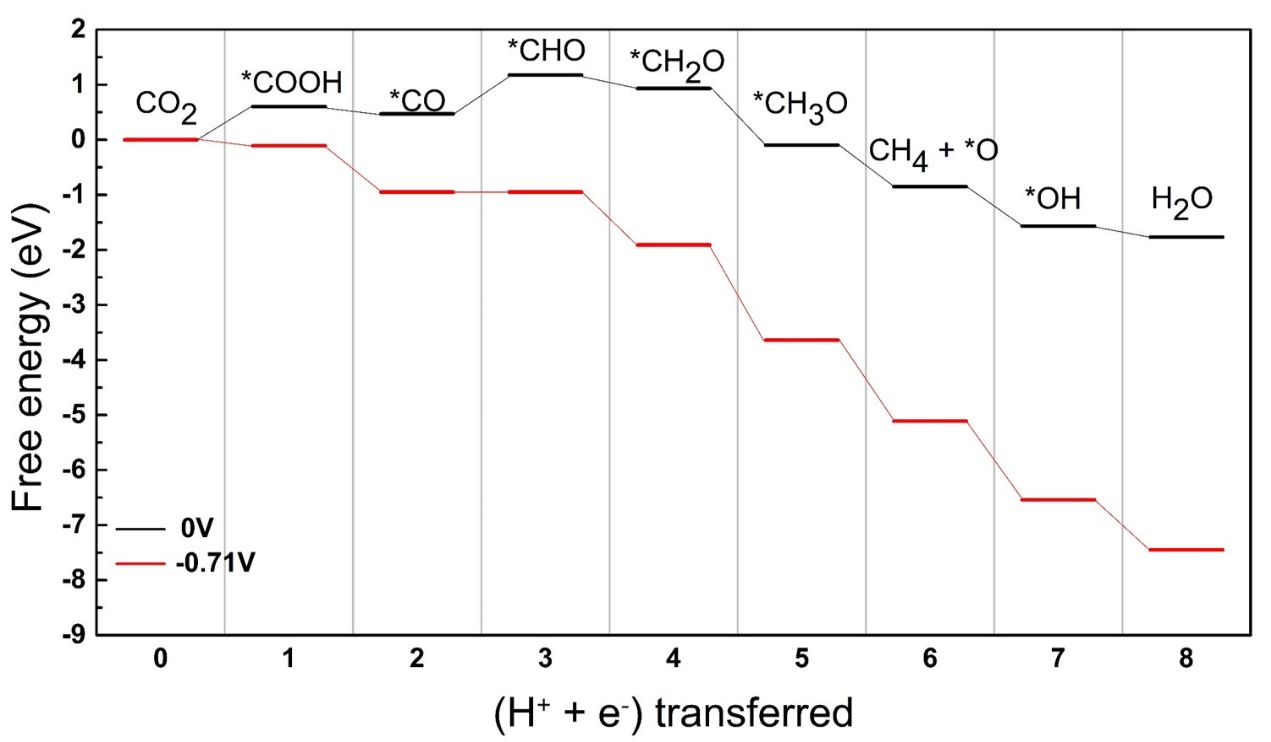


**Fig. S6.** Free energy diagrams for the lowest energy pathways to CH_4_ on In_4_Cu_9_. The black (higher) pathway represents the free energy at 0 V *vs.* RHE and the red (lower) pathway the free energy at the indicated potential.


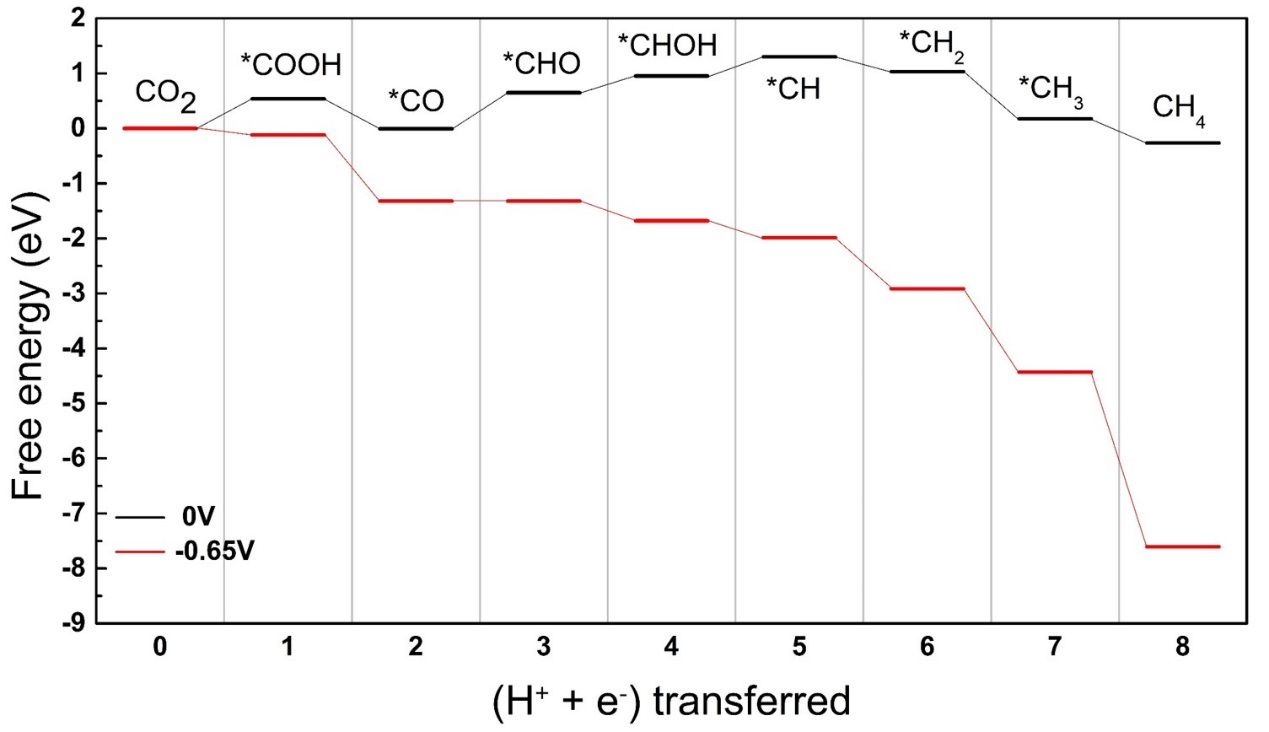


**Fig. S7.** Free energy diagrams for the lowest energy pathways to CH_4_ on InCu_2_. The black (higher) pathway represents the free energy at 0 V *vs.* RHE and the red (lower) pathway the free energy at the indicated potential.


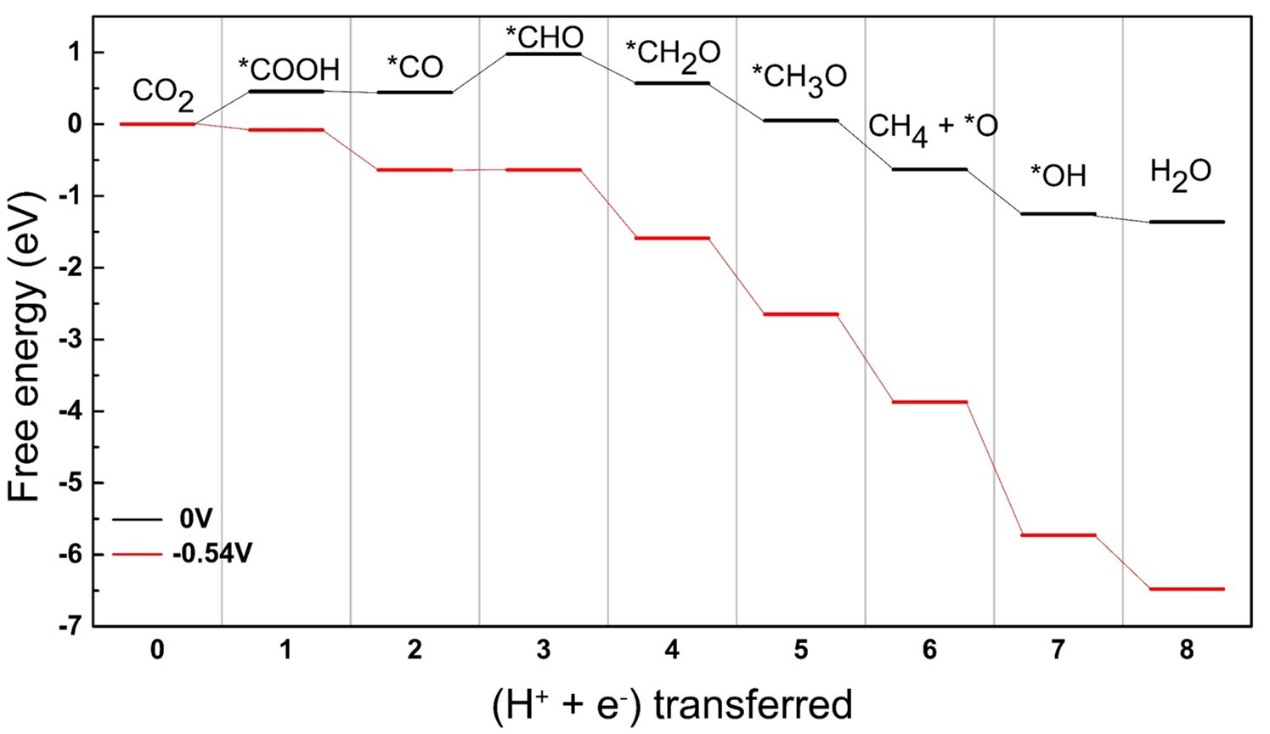


**Fig. S8.** Free energy diagrams for the lowest energy pathways to CH_4_ on In_7_Cu_10_. The black (higher) pathway represents the free energy at 0 V *vs.* RHE and the red (lower) pathway the free energy at the indicated potential.


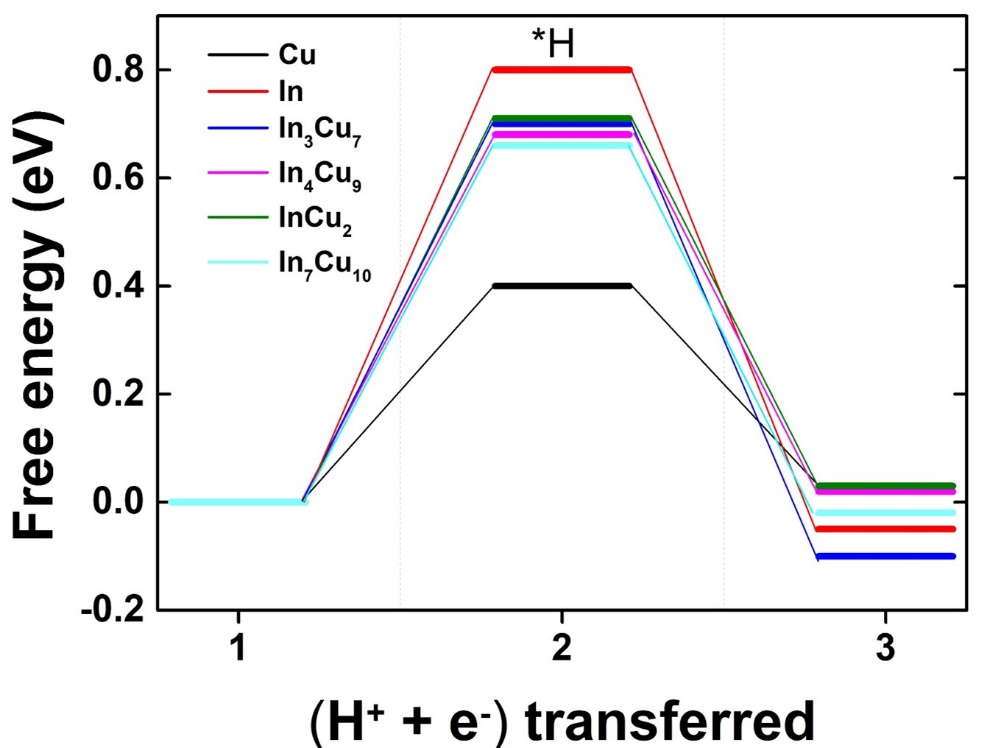


**Fig. S9.** Free energy diagrams for the lowest energy pathways to H_2_ for Cu metal, In metal, In_3_Cu_7_, In_4_Cu_9_, InCu_2_ and In_7_Cu_10_.
